# Application of ICHD-II Criteria in a Headache Clinic of China

**DOI:** 10.1371/journal.pone.0050898

**Published:** 2012-12-11

**Authors:** Zhao Dong, Hai Di, Wei Dai, Jingyao Liang, Meiyan Pan, Mingjie Zhang, Zhibin Zhou, Zheng Li, Ruozhuo Liu, Shengyuan Yu

**Affiliations:** International Headache Center, Department of Neurology, Chinese PLA General Hospital, Beijing, China; Torrey Pines Institute for Molecular Studies, United States of America

## Abstract

**Background:**

China has the huge map and the largest population in the world. Previous studies on the prevalence and classification of headaches were conducted based on the general population, however, similar studies among the Chinese outpatient population are scarce. This study aimed to analyze the characteristics of 1843 headache patients enrolled in a North China headache clinic of the General Hospital for Chinese People's Liberation Army from October 2011 to May 2012, with the International Classification of Headache Disorders, 2nd Edition (ICHD-II).

**Methods and Results:**

Personal interviews were carried out and a detailed questionnaire was used to collect medical records including age, sex and headache characteristics. Patients came from 28 regions of China with the median age of 40.9 (9–80) years and the female/male ratio of 1.67/1. The primary headaches (78.4%) were classified as the following: migraine (39.1%), tension-type headache (32.5%), trigeminal autonomic cephalalgias (5.3%) and other primary headache (1.5%). Among the rest patients, 12.9% were secondary headaches, 5.9% were cranial neuralgias and 2.5% were unspecified or not elsewhere classified. Fourteen point nine percent (275/1843) were given an additional diagnosis of chronic daily headache, including medication-overuse headache (MOH, 49.5%), chronic tension-type headache (CTTH, 32.7%) and chronic migraine (CM, 13.5%). The visual analogue scale (VAS) score of TTH with MOH was significantly higher than that of CTTH (6.8±2.0 vs 5.6±2.0, *P<*0.001). The similar result was also observed in VAS score between migraine with MOH and CM (8.0±1.5 vs 7.0±1.5, *P* = 0.004). The peak age at onset of TTH for male and female were both in the 3^rd^ decade of life. However, the age distribution at onset of migraine shows an obvious sex difference, i.e. the 2^nd^ decade for females and the 1^st^ decade for males.

**Conclusions/Significance:**

This study revealed the characteristics of the headache clinic outpatients in a tertiary hospital of North China that migraine is the most common diagnosis. Furthermore, most headaches in this patient population can be classified using ICHD-II criteria.

## Introduction

The global prevalence of active headache disorders in the adult population is 46% in general, 11% for migraine, and 42% for tension-type headache (TTH), respectively [1]. In both primary care units and neurological clinics, headache disorder is considered to be one of the most common causes of medical consultation [2], [3]. However, most headaches can be self-managed and do not require further medical intervention. Thus, headache patients visiting clinic may show different clinical features and types from those obtained from the general population-based studies. TTH was reported to be the most frequent diagnosis, followed by secondary headache and migraine in the emergent department based on a Greek study [4]. However, in a Hungary study, migraine was the most common headache, while TTH patients reported more severe disability [5]. Studies from Spain and Pakistan also indicated that migraine was the most common diagnosis [6], [7].

Although China has the largest headache population (over 1.3 billion), clinical studies on the headache disorders are scarce. This is largely due to the lack of applying unified clinic criteria for the classification and diagnosis of headache disorders. A most recent study focusing on the Chinese headache disorders was conducted in a neurological clinic from a southwest tertiary hospital, which showed that migraine was the most common headache diagnosis in neurological services. The study suggested that more attention should be paid to the intensity in migraine patients, and the frequency of TTH as well [3]. However, China is a large country with different ethnics who viewed headache in different ways and had varied tolerabilities to headache disorders. Meanwhile, the regional difference also contributes to the different view of headaches.

The purpose of the current study was to analyze the incidence and characteristics of the different types of headache with the strict application of the International Classification of Headache Disorders, 2nd edition (ICHD-II) [8] in a headache clinic from a tertiary hospital of North China.

## Methods

Retrospective review was performed based on the medical records of headache patients who presented to a headache clinic of the General Hospital for the People's Liberation Army (PLA) of China from October 2011 to May 2012. The hospital is a tertiary care center in Beijing that serves patients mostly from the North China regions including Beijing, Hebei, Inner Mongolia, Shanxi, Henan, Shandong, Heilongjiang, Shaanxi, Jilin and Liaoning, et al. For each patient, a routine questionnaire was completed by a qualified and experienced specialist in headache neurology. The questionnaire is comprised of demographic data, headache profile (location, duration, attack frequency, severity, accompanying symptoms, aura, causative and relieving factors), previous prophylaxes, treatment and family history. ICHD-II was strictly followed to classify the patients into 14 different subgroups. Subgroups 1 to 4 were primary headaches including migraine, TTH, cluster headache and others. Subgroups 5 to 12 were secondary headaches which are consequences of head and/or neck trauma, cranial and cervical vascular disorder, non-vascular intracranial disorder, substance abuse or withdrawal, infection, homoeostatic disorder, disorder of facial or cranial structural tertiary and psychiatric disorders. Subgroup 13 represented cranial neuralgias and facial pain with central causes. If the headache could not be accurately categorized as either primary or secondary, it was classified as subgroup 14 (unspecified or not elsewhere classified headaches).

Patients who reported headaches at a frequency of more than 15 days/month over a period of 3 months were classified as chronic daily headache (CDH) [9]. CDH includes chronic migraine (CM), chronic tension-type headache (CTTH), medication-overuse headache (MOH) which presents as migraine or TTH, new daily persistent headache (NDPH), and hemicrania continua (HC). MOH patients were followed up in a 2-months period in order to confirm the diagnosis. Patients who fulfilled criteria for more than 1 type of headache were categorized based on their “most troublesome headache” at the time of clinic visit.

The study protocol was approved by the Ethical Committee of Chinese PLA General Hospital and complied with the Declaration of Helsinki. The informed consents were obtained from all patients before the study.

All data were expressed as mean ± SD and the descriptive statistics were used. The Student's *t*-test or one-way analysis of variance (ANOVA) was used to compare the continuous variables. SPSS for windows (Version 18.0) software was used for statistical analyses. All calculated *P*-values were 2-tailed and statistical significance was defined as a *P* value<0.05.

## Results

A total of 1843 patients (1152 female) attended our headache clinic. The median age was 40.9 years (range: 9–80 years).

**Figure 1:**
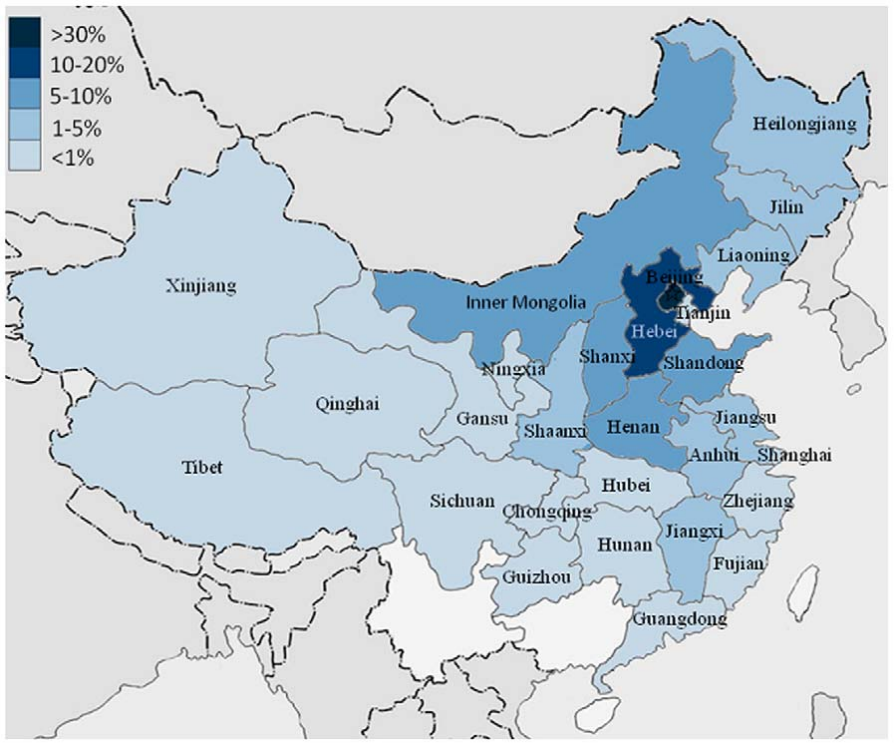
Regional distribution of headache patients in the current study (n = 1843). Patients came from 28 regions of China. Colors from dark to light indicate different proportion of headache patients from higher to lower (5 levels: >30%, 10–20%, 5–10%, 1–5%, and <1%).

Patients came from 28 regions of China (Figure 1). Most of them live in North China area including Beijing (606, 32.9%),Hebei (312, 16.9%), Inner Mongolia (183, 9.9%), Shanxi (134, 7.3%),Henan (132, 7.2%),Shandong (109, 5.9%), Heilongjiang (66, 3.6%), Liaoning (41, 2.2%), Shaanxi (40, 2.2%), Jilin (40, 2.2%), Anhui (31, 1.7%). Other areas included Jiangsu (21, 1.1%), Jiangxi (18, 1.0%), Hunan (17, 0.9%), Sichuan (17, 0.9%), Hubei (14, 0.8%), Tianjin (13, 0.7%), Gansu (10, 0.5%), Xinjiang (7, 0.4%), Fujian (7, 0.4%), Guangdong (6, 0.3%), Guizhou (5, 0.3%), Chongqing (4, 0.2%), Ningxia (2, 0.1%), Zhejiang (2, 0.1%), Shanghai (2, 0.1%), Qinghai (1, 0.05%), and Tibet (1, 0.05%). Other two patients came from America and India respectively.

**Figure 2:**
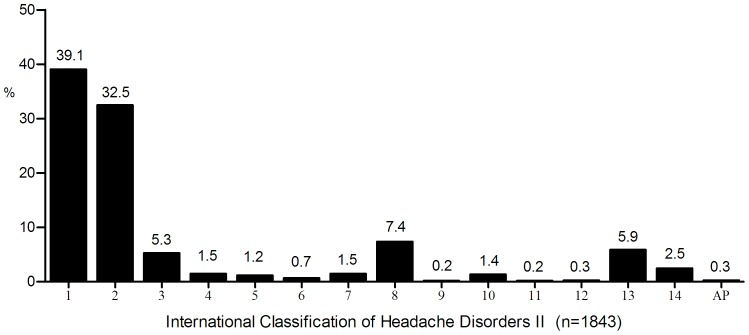
Headache patients (n = 1843) were diagnosed according to ICHD II classification. Groups 1 to 4 indicated 4 types of primary headaches of ICHD-II (including migraine, TTH, trigeminal autonomic cephalgias, and other primary headaches) and groups 5 to 12 indicated secondary headaches. Group 13 and 14 indicated cranial neuralgias and central causes of facial pain, and unspecified or not elsewhere classified headaches, respectively. (Ap = appendix).

Of all the headache patients in this study, 78.4% were diagnosed as primary headaches (groups 1 to 4 of ICHD-II) and 12.9% were secondary headaches (groups 5 to 12). Of the remained patients, 5.9% were classified as group 13 and 2.5% as group 14. There was a low incidence of secondary headaches in our series: 22 (1.2%) cases were classified as group 5 (Headaches attributed to trauma), 13 (0.7%) as group 6 (Headache attributed to cranial or cervical vascular disorder), 28 (1.5%) as group 7 (Headache attributed to non-vascular intracranial disorder), 137 (7.4%) as group 8 (Headaches attributed to a substance abuse or withdrawal) which included 136 MOH, 3 (0.2%) as group 9 (Headache attributed to infection), 26 (1.4%) as group 10 (Headache attributed to disorder of homeostasis), 3 (0.2%) as group 11 (Headache or facial pain attributed to disorder of cranium, neck, eyes, ears, nose, sinuses, teeth, mouth or other facial or cranial structures), and 5 (0.3%) as group 12 (Headache attributed to psychiatric disorder). Other 109 (5.9%) cases were classified as group 13 (Cranial neuralgias and central causes of facial pain). Only 46 patients (2.5%) could not be classified into any group according to ICHD-II criteria, and were thus listed as group 14 (Unspecified or not elsewhere classified headaches). The percentages in each group were shown in Fig. 2.

**Figure 3:**
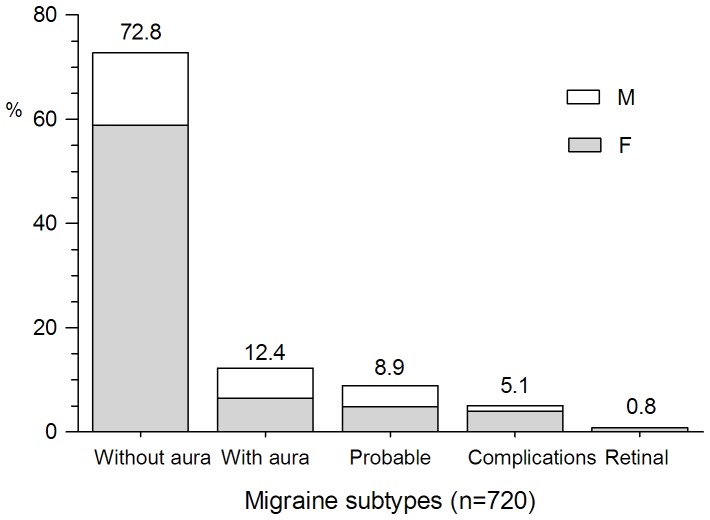
Percentage of migraine subtypes. Migraine without aura was the most common subtype, followed by migraine with aura, probable migraine, migraine complications, and retinal migraine.

**Figure 4:**
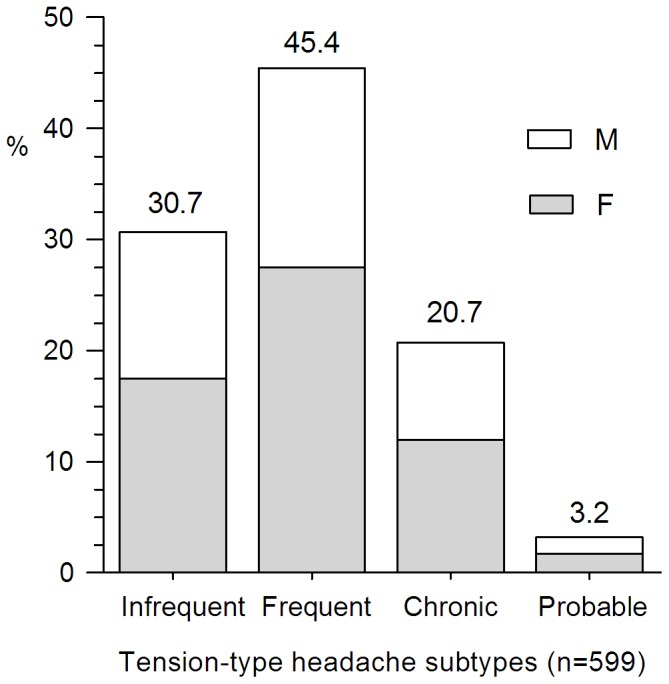
Percentage of TTH subtypes. Frequent episodic TTH was the most usually diagnosed TTH type in current study. Infrequent episodic was the second most common TTH type, followed by CTTH.

**Figure 5:**
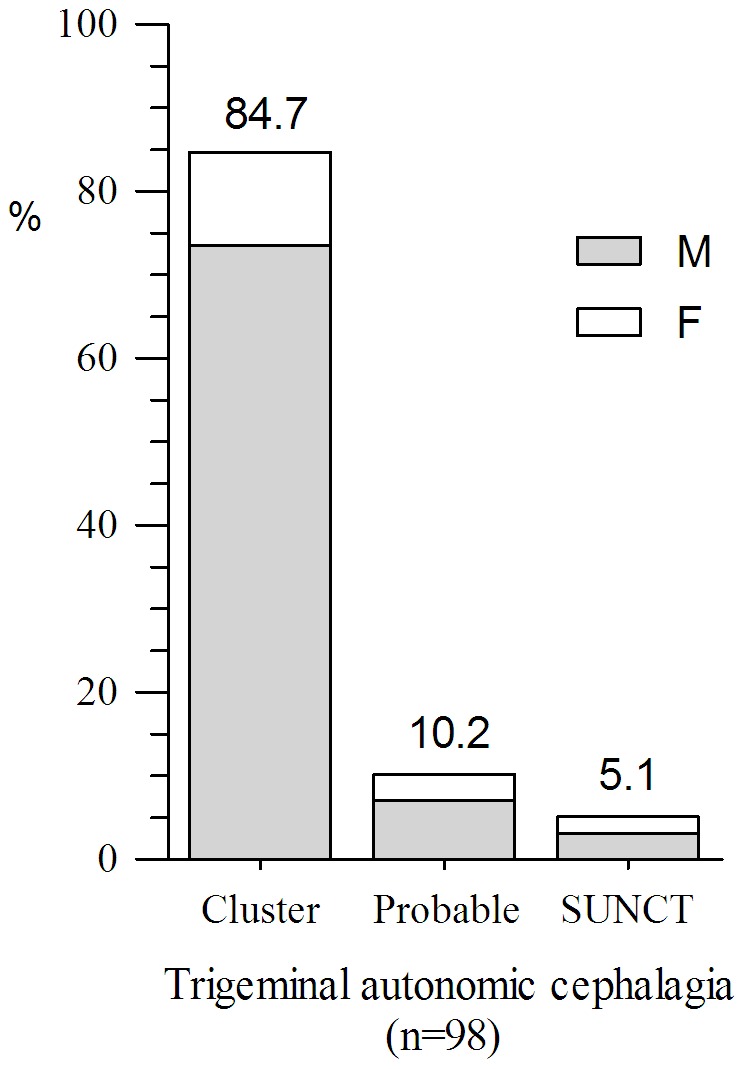
Percentage of trigeminal autonomic cephalagia subtypes. Cluster headache was the commonest subtype including 72 male and 11 female. The Remained 15 cases consisted of 10 probable cluster headache and 5 SUNCT. Paroxysmal hemicranias patients were not found in current study. (SUNCT = short-lasting unilateral neuralgiform headache with conjunctival injection and tearing).

With regard to primary headaches, 39.1% (720/1843) were migraine. Seventy-two point eight percent (524/720) of migraines (female: male, 58.9%: 13.9%) fulfilled the diagnostic criteria of migraine without aura and 12.4% (89/720) of migraineurs (female: male, 6.5%: 5.8%) were with aura. TTH accounted for 32.5% (599/1, 843) of the total patients. Frequent episodic TTH (45.4%, 272/599) was the most usually diagnosed TTH type including 27.5% of female and 17.9% of male. Infrequent episodic (30.7%, 184/599) was the second most common TTH type, followed by CTTH (20.7%, 124/599). Ninety-eight patients (5.3%) were diagnosed as trigeminal autonomic cephalgias, and the cluster headache was the commonest subtype including 72 (73.5%) male and 11 (11.2%) female. The remaining 15 cases consisted of 10 probable cluster headache (7 cases for lacking autonomic features and 3 cases for longer than 3 hours) and 5 short-lasting unilateral neuralgiform headache with conjunctival injection and tearing (SUNCT). Paroxysmal hemicranias patients were not found in our study. The gender ratios of different primary headache subtype are shown in Figs. 3, 4, 5.

**Figure 6:**
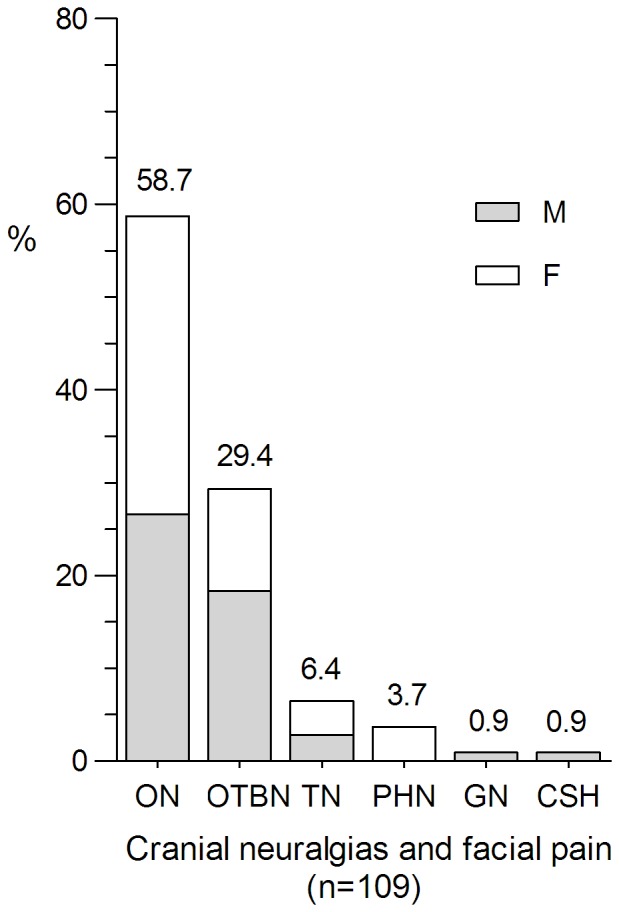
Percentage of cranial neuralgias and facial pain subtypes. ON was the most common subtype, followed by OTBNs, TN, PHN, GN and GSH. (ON = occipital neuralgia. OTBN = other terminal branch neuralgias. TN = trigeminal neuralgia. PHN = post-herpetic neuralgia. GN = glossopharyngeal neuralgia. GSH = cold-stimulus headache).

With regard to group 13 (cranial neuralgias and central causes of facial pain), occipital neuralgia (58.7%, 64/109) was the most common subtype, followed by other terminal branch neuralgias (29.4%, 32/109), trigeminal neuralgia (6.4%, 7/109), post-herpetic neuralgia (3.7%, 4/109), glossopharyngeal neuralgia (0.9%, 1/109) and cold-stimulus headache (0.9%, 1/109). The gender ratios are similar in these groups (50.5 vs. 49.5%). The gender ratios of different subtypes are shown in Fig. 6.

**Figure 7:**
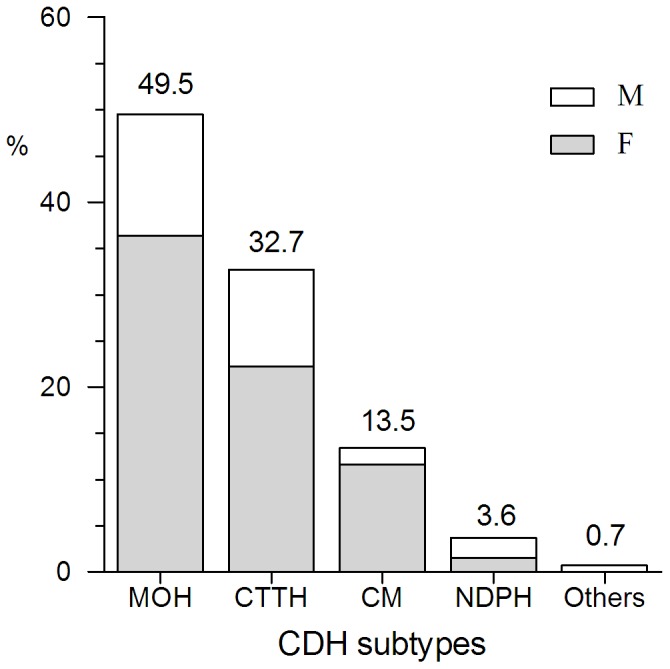
Percentage of CDH subtypes (total, 275 cases). MOH was the most common CDH which consist of 48 TTH patients and 88 migraineurs, followed by CTTH, CM and NDPH. (CDH = chronic daily headache, MOH = medication overuse headache, CTTH = chronic tension-type headache, CM = chronic migraine, NDPH = new daily persistent headache).

**Figure 8:**
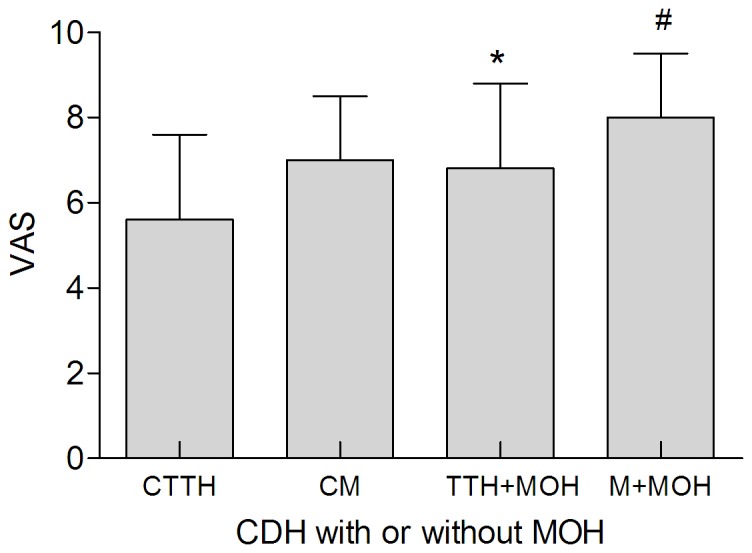
The relationship between VAS score and CDH subtypes. * Compared with CTTH, P<0.001, # compared with CM, P = 0.004. (CDH = chronic daily headache, CTTH = chronic tension-type headache, CM = chronic migraine, MOH = medication overused headache, M = migraine).

Of the total patients, 14.9% (275/1843) were given an additional diagnosis of CDH. As shown in Fig. 7, MOH (49.5%, 136/275) was the most common CDH which consist of 48 TTH patients and 88 migraineurs, followed by CTTH (32.7%, 90/275), CM (13.5%, 37/275), NDPH (3.6%, 10/275). Two patients (0.7%, both male) cannot be diagnosed with proper CDH subtypes and classified to other CDH. Females outnumbered males among the CDH patients (71.6 vs. 28.4%). The gender ratios of different CDH subtype are shown in Fig. 7. The VAS scores for CTTH, CM, TTH with MOH and migraine with MOH were 5.6±2.0 (n = 90), 7.0±1.5 (n = 37), 6.8±2.0 (n = 48), and 8.0±1.5 (n = 88) respectively (Fig. 8). There was a significant difference among four groups (F = 26.90, *P<*0.001). The VAS score of TTH with MOH was significantly higher than that of CTTH (*P<*0.001). The similar result was also observed in VAS score between CM and migraine with MOH (*P* = 0.004).

**Figure 9:**
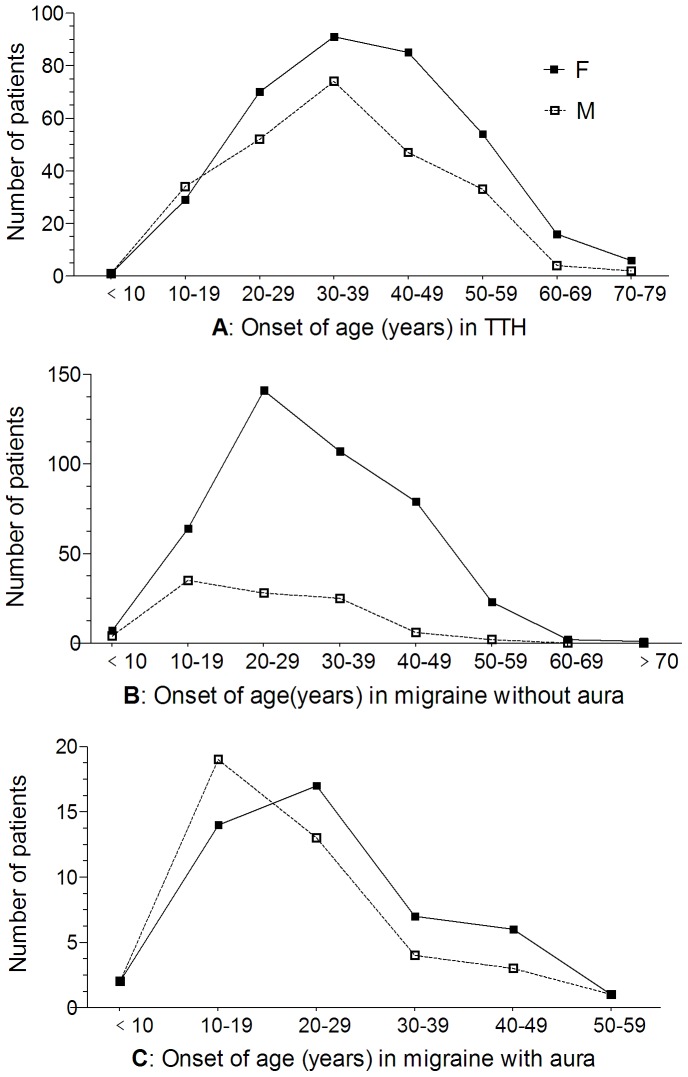
Age distribution at onset for TTH (A), migraine without (B) or with aura (C). A: The peak age at onset for male and female were both in the 3^rd^ decade of life for TTH patients. B–C: The summit period of migraine, both without aura and with aura, seem to advance to 2^nd^ for female and 1^st^ for male.

The onset age distributions patterns for TTH and migraine are shown in Figs. 9. For TTH (Fig. 9A), the peak age at onset for male and female were both in the 3^rd^ decade of life. However, the age distributions of migraine, with (Fig. 9B) and without (Fig. 9C) aura, show an obvious sex difference. In female, there was a significant peak age in the 2^nd^ decade for females. On the contrary, the number of males peaked at the 1^st^ decade, and then gradually decreased after 20 years old.

## Discussion

China has the huge map and the largest population in the world. The estimated 1-year prevalence of primary headache in China was 23.8%, including migraine (9.3%) and TTH (10.8%) [10]. Previous study on the prevalence and classification of headaches was conducted based on the general population [10], however, similar study of headache disorders in the outpatient population is scarce in China. Although the classification of headache were conducted in a study on neurological outpatients [3], our study offers some new merits to this field. First, ICHD-2 is strictly applied in the study and every headache patient was assigned to a specific subtype. Second, we conducted our study based on patients from a headache clinic which might offer patients with more severe headache symptoms than those in the general neurological outpatients. Third, most patients who attended our hospital were from North China area (Fig. 1), which is different from study in Southwest area of China. Previous Japanese study has reported a regional difference in the prevalence of migraine and speculated a genetic factor as the cause of regional difference in migraine prevalence [11]. It is still unknown whether there are differences between above-mentioned two regions in China.

Our study indicated that migraine and TTH were the two most common presentations, accounting for 39.1% and 32.5% of the total patients, respectively. This is consistent with the previous studies [3], [5]–[7], [12], [13]. Although it is suggested that TTH is the most common type of primary headache all over the world [1], migraine seemed to be the most common presentation in our series, accounting for 39.1% of total headache patients. This percentage is similar to the previous publications from the headache clinics [5], [6] and a tertiary care center in Brazil (migraine, 38%; TTH, 22.6%; and cluster headache, 2.7%) [14], but higher than those obtained from neurological and emergency departments from China [3] and US [15]. Several possible explanations include under-recognition of TTH by patients and health practitioners for its less disability than migraine and lower percentage of medical referral from neurological and other department.

Although cluster headache and other trigeminal autonomic headaches (group 3) are much less common than other severe headaches such as migraine and TTH, 5.3% of total patients were classified into group 3 in our study, which was higher than that obtained from Southwest China (0.5%) [3] and Spain (2.6%) [6]. The reasonable explanation may be the fact that cluster headache is an excruciating headache syndrome and patients often responded poor to nonspecific treatment such as analgesics and the low recognition rate by many physicians and even neurologist. As a result, these patients are willing to go to headache clinic for specific treatment. In addition to cluster headaches, other trigeminal autonomic headaches included 5 cases with SUNCT. Yet, paroxysmal hemicranias is absent in our series. The fact might be attributed to regional and racial reasons. For cluster headache, the male: female ratio was 6.5∶1, which was similar to results from Taiwanese (6.4∶1) [16] and Japanese (3.8∶1) [17].

The percentage of secondary headache (groups 5 to 12) is lower (13.1%) in this study than that reported by Wang (27%) [3], but similar to the results of Guerrero et al. (14.3%) [6]. However, other studies from emergency departments and general practice patients revealed higher percentage of secondary headaches ranging from 22.1% to 42% [4], [15], [18], [19]. We speculated that the reason is that physicians from both neurological and emergency department outpatient are trained to recognize headache disorders as diseases that were attributed to some somatic reasons. Therefore, patients suffered from primary headache disorders are more likely to go to headache clinic with negative neuroimaging results and recurrent attacks.

Fourteen point nine percent of patients were diagnosed as CDH. The percentage is similar to another report from Wang (11.4%, 192/1683) from southwest China [3], but lower than those reported by Murtaza (39%) in Pakistan and Chakravarty (50%) in India [7], [20]. The reason may be the relatively lower prevalence of CDH (1.0%) in China [10] than other regions of the world (3% to 5%) [21]. MOH is the most common CDH, accounting for about 49.5% (136/275) of total patients. It is also the most common subtype in group 8 (Headaches attributed to a substance abuse or withdrawal). The result is consistent with the one reported by Guerrero [6] but different from the following ones. Wang et al indicated that TTH was the most prevalent CDH subtype [3], while Murtaza, Chakravarty and Lu found that CM was more popular [6], [20], [22]. The VAS scores of TTH and migraine, with or without MOH, were compared and the results indicated that both TTH and migraine with MOH have significantly higher VAS scores than those without MOH. It is known that medication overuse does appear to be a significant risk factor in converting frequent episodic headache into CDH [23]. However, our results suggested that medication overuse may also aggravate the pain intensity, resulting in gradually deteriorative quality of life for patients.

The peak ages of TTH at onset for male and female were both in the 30–39 years in the current study. But the summit period of migraine seems to advance to 20–29 for female and 10–19 for male. A recent study from US population suggested a bimodal distribution of migraine prevalence, showing that the peak periods for migraine risk were at a mean age of 25 years and 50 years in women but a little earlier in men [24]. Our study was consistent with these previous reports for the first but not the second peak period, suggesting that there may be some racial, regional, selection bias, methodological factors that contributed to these differences.

There are some limitations for the current study. First, our data present one selecting bias, as these patients suffered more frequent and severe headaches than the general population. Also, the sample of the study has been collected only in one headache centre and therefore may present another epidemiologically selecting bias. Last, we compared only a limited number of clinical migraine cases especially with aura, and further studies are needed.

In conclusion, the large majority of headaches in our series could be classified according to ICHD-II criteria. Although our registry is heterogeneous and includes different sources of referral, it shows the distribution of different headache disorders seen in a headache clinic of China.
